# Congenital gastrofundic jejunal fistula in an adult: a case report

**DOI:** 10.1093/jscr/rjag624

**Published:** 2026-11-10

**Authors:** Yi-Ming Huang, Shen-Hao Xu, Hai-Lin Gan, Jing Yu, Wen-Hai Fan, Xiulian Xu, Zhi-Heng Liu, Pan Wang

**Affiliations:** Department of Gastrointestinal Surgery, Affiliated Hospital of North Sichuan Medical College, Nanchong 637000, China; Department of Gastrointestinal Surgery, Affiliated Hospital of North Sichuan Medical College, Nanchong 637000, China; Department of Gastrointestinal Surgery, Affiliated Hospital of North Sichuan Medical College, Nanchong 637000, China; Department of Gastrointestinal Surgery, Nanchong Central Hospital Affiliated to North Sichuan Medical College, Nanchong 637000, China; Department of Gastrointestinal Surgery, Affiliated Hospital of North Sichuan Medical College, Nanchong 637000, China; Department of Gastrointestinal Surgery, Affiliated Hospital of North Sichuan Medical College, Nanchong 637000, China; Department of Gastrointestinal Surgery, Affiliated Hospital of North Sichuan Medical College, Nanchong 637000, China; Department of Gastrointestinal Surgery, Affiliated Hospital of North Sichuan Medical College, Nanchong 637000, China

**Keywords:** congenital, Gastrofundic jejunal fistula, gastrointestinal malformation, adult, laparoscopic surgery

## Abstract

Gastrofundic jejunal fistula is a rare but clinically severe condition characterized by an abnormal communication between the gastric fundus and jejunum, allowing gastric contents to bypass the normal gastrointestinal pathway. Most cases are documented on secondary gastrojejunal fistula due to peptic ulcer disease, malignancy, or postoperative complications. Here, we report an extremely rare case of congenital gastrofundic jejunal fistula in a 56-year-old male who present with postprandial abdominal pain for 6 months. After multidisciplinary team evaluation, the patient underwent laparoscopy combined with gastroscopy for localizing the fistula. The abnormal fistulous tract was identified and surgically treated. Postoperative recovery was uneventful, and no adverse events or specific complaints were noted during the 6-month follow-up period.

## Introduction

Gastrofundic jejunal fistula is an abnormal anatomical communication between the gastric fundus and the jejunum; due to the rarity of this disease, it has rarely been reported on. Although uncommon in clinical practice, it may lead to severe malabsorption, electrolyte imbalance, and nutritional deficiencies [1]. Previous reported cases are mostly secondary to acquired conditions, particularly peptic ulcer disease, malignancy, or postoperative complications. As early as 1936, Scrimger [2] firstly described the surgical techniques for managing gastrojejunal ulcers complicated by fistula formation. Subsequent studies confirmed that spontaneous gastrojejunal fistula can occur as a complication of gastric ulcer disease [3–5]. Malignant infiltration is another recognized etiology, and some researchers have documented a case in which gastric cancer penetrated the jejunum, allowing contrast material to pass directly into the small intestine [6]. In addition to these causes, rare iatrogenic factors have been described. For example, gastrojejunal fistula formation has been reported following gastrostomy tube placement [7]. However, gastrofundic jejunal fistula is a extremely rare condition. To the best of our knowledge, this is the first case reported in the literature of congenital gastrofundic jejunal fistula in an adult. In this article, we describe the anatomical variation of this case and discuss the diagnosis and treatment of a patient with congenital gastrofundic jejunal fistula.

## Case presentation

A 56-year-old male was admitted to our hospital on August 17, 2025, with a 6-month history of postprandial abdominal pain. Before his admission, the patient was evaluated at a local hospital, where contrast-enhanced abdominal computed tomography (CT) suggested possible intestinal adhesions with mild dilation of the proximal intestine and stomach, as well as a suspected gastrojejunal fistula. Despite receiving conservative treatment, the abdominal pain persisted without significant improvement. The patient had no history of foreign body ingestion (such as fruit pits or animal bones), peptic ulcer disease, gastric cancer, or prior abdominal surgery.

Physical examination revealed persistent dull pain and localized tenderness in the mid-upper abdomen, without rebound tenderness or abdominal muscle guarding. The remainder of the abdominal examination was normal. The patient’s vital signs were as follows: body temperature 36.4°C, pulse 75 beats/min, respiratory rate 18 breaths/min, and blood pressure 131/80 mmHg. Routine blood examination revealed the following: white blood cell count of 4970/mm^3^ (normal range 4000–10 000/mm^3^), proportion of neutrophils of 50.7% (normal range 40–75%), red blood cell count of 4900/mm^3^ (normal range 3800–5100/mm^3^), and platelet count of 184 × 10^3^/mm^3^ (normal range 100–300/mm^3^). Liver and renal function tests were within normal limits.

An upper digestive tract ioversol angiography showed a normotonic stomach with early opacification of the jejunum, demonstrating a fistulous tract between the gastric fundus and the jejunum. (Fig. 1A and B). Abdominal enhanced CT scan showed a focal disruption of the gastric wall along the greater curvature of the gastric fundus, which communicated with the underlying proximal jejunum via a visible fistulous tract (Fig. 1C). Gastroscopy revealed a depression on the posterior wall of the gastric fundus adjacent to the greater curvature, surrounded by converging gastric folds, indicating a fistulous tract formation (Fig. 1D). Based on the symptoms and the above imaging findings, the diagnosis of a congenital gastrofundic jejunal fistula was suspected.

**Figure 1:**
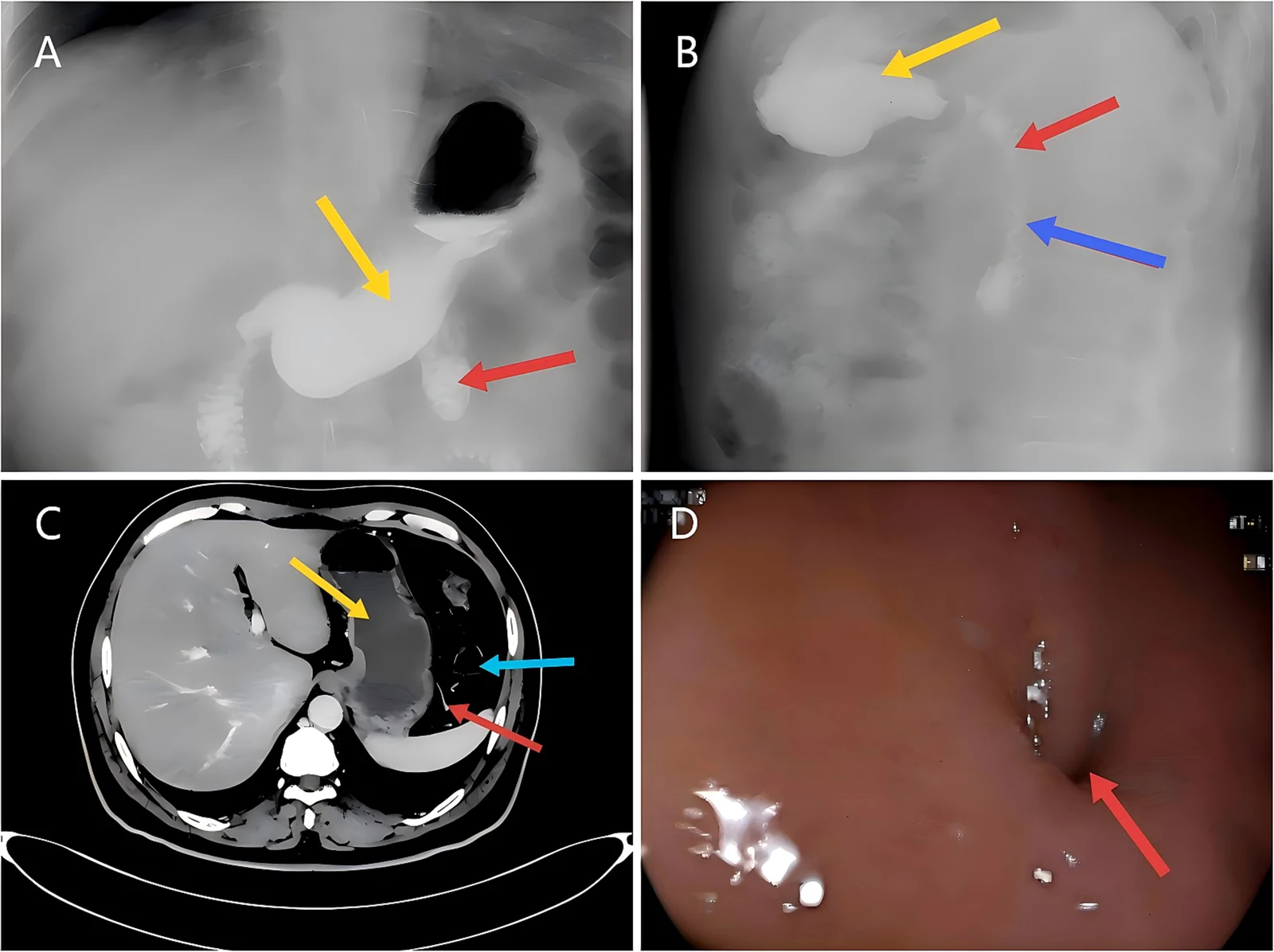
Imaging and endoscopic findings. (A, B) Upper gastrointestinal soluble iodinated oil contrast imaging (anteroposterior and lateral views) showed a medium filling in the gastric body with contrast agent (yellow arrow), with contrast passing from the greater curvature of the gastric fundus downward through the fistula tract (red arrow) into the jejunum (blue arrow); (C) Enhanced abdominal CT revealed a well-defined gastric body imaging (yellow arrow), with a tubular channel at the greater curvature of the gastric fundus communicating with the posterior jejunum (red arrow) and the distal inferior jejunum (blue arrow); (D) Gastroscopic examination indicated a circumferential concentric gastric fold formation on the greater curvature side of the gastric wall, suggesting the possibility of a fistula tract (red arrow).

Following multidisciplinary team discussion and comprehensive preoperative preparation, laparoscopic exploration was performed for the patient. Intraoperatively, due to severe adhesions in the upper abdominal cavity, the fistulous tract could not be identified; therefore, the gastroscopy was underwent during the operation procedure for this case, which successfully revealed the fistula. Additionally, significant anatomical variations were identified in the patient’s abdominal cavity as follows. The ligament of Treitz was found to be absent; a segment of jejunum, approximately 10 cm in length, ascended through the transverse mesocolon and coursed posterior to the pancreas, communicating with the posterior wall of the gastric fundus near the greater curvature, forming a fistulous tract with severe adhesions to the surrounding tissues (Fig. 2A). After separating the fistula tube, an endoscopic linear cutting stapler (EC60A, Ethicon) was used to for the resection and closure of the fistula at the gastric fundus (Fig. 2B). Finally, a drainage tube was routinely placed in the abdominal cavity. The operation time was 128 min and intraoperative blood loss was 72 ml.

**Figure 2:**
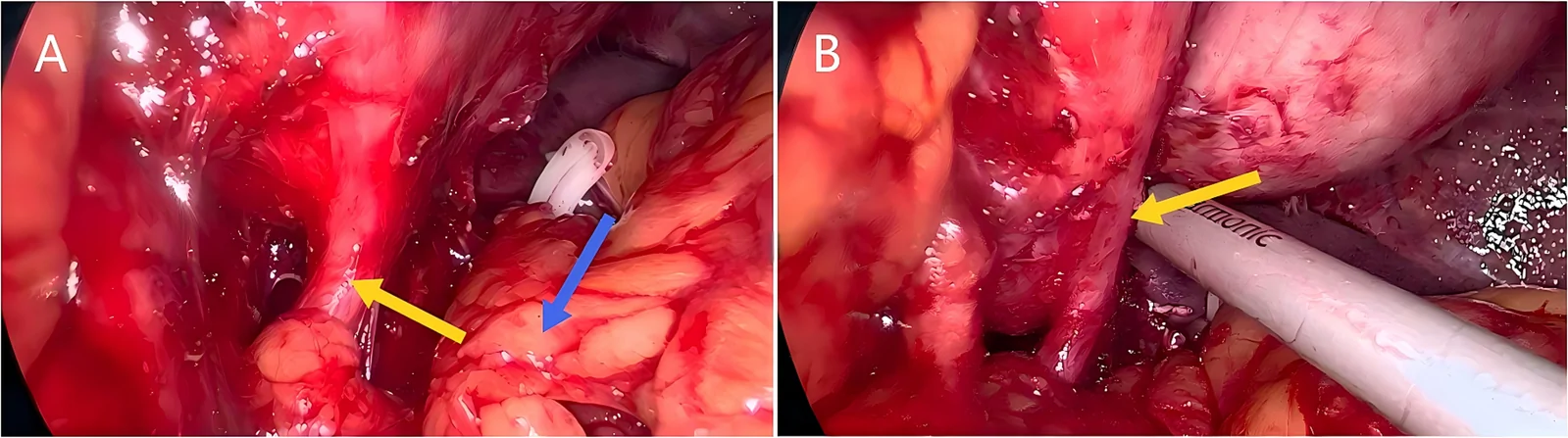
Laparoscopic exploration results. (A) Showing the fistula tract (yellow arrow) connecting to the greater curvature of the gastric fundus posterior to the pancreas (blue arrow); (B) After dissection of the fistula, a linear stapler (EC60A, Ethicon) was used to transect the fistulous tract and close the fistula (yellow arrow).

The patient had an unremarkable postoperative recovery. Oral feeding was initiated on postoperative day 3 after flatus, and no epigastric pain or other symptoms were mentioned. Subsequently, the patient was discharged on postoperative day 5. During the 6-month follow-up period, no specific symptoms or discomforts were reported, and the findings of upper gastrointestinal radiography and gastroscopy examination were normal (data not shown).

## Discussion

It is well established that congenital gastrointestinal anomalies are extremely rare in adults. A congenital gastrojejunal fistula is an abnormal passage connecting the stomach and proximal jejunum that arises during embryonic development. Unlike acquired fistulas, this condition results from a congenital malformation of the digestive tract, forming a persistent abnormal channel between the stomach and jejunum [8]. Here, we report the diagnosis and management of a 56-year-old male with congenital gastrofundic jejunal fistula, aiming to improve clinical awareness and provide insights into diagnostic and therapeutic strategies for this rare condition.

It has been elucidated that typical symptoms of congenital gastro-jejunal fistula may include nausea, vomiting, weight loss, diarrhea, and cramp-like abdominal pain. These manifestations may result in severe complications such as metabolic disturbances, vitamin deficiencies, malnutrition, and even multi-organ dysfunction [9]. Based on the preoperative gastroscopic findings (a depression on the posterior wall of the gastric fundus near the greater curvature with converging gastric folds, a typical sign of a fistulous orifice) and the intraoperative laparoscopic findings (the fistulous tract was observed to emerge from behind the pancreas and communicate with the adjacent jejunum), no evidence of other primary diseases such as peptic ulcer, malignancy, trauma, or prior abdominal surgery was identified. Therefore, this case was considered a congenital gastrofundic jejunal fistula. However, the adult patient with gastrofundic jejunal fistula reported in this article did not present with the aforementioned symptoms, only experiencing postprandial upper abdominal pain in the last 6 months, which had a relatively minor impact on the patient.

Imaging examination is the primary method for the diagnosis of gastrofundic jejunal fistula. Upper gastrointestinal contrast examination can demonstrate contrast agent passing directly from the stomach into the jejunum. When a gastrofundic jejunal fistula is identified in adults, the vital objective is to differentiate between congenital and acquired causes (the latter often resulting from gastric ulcers, malignant tumors, trauma, or iatrogenic injury), which requires the use of gastroscopy, endoscopic ultrasound, CT, and other imaging examinations [10]. Presently, in addition to the upper gastrointestinal contrast examination for this case, we further performed gastroscopy and CT examinations to rule out secondary factors such as gastric ulcers, tumors, and injuries, which can provide the correct direction for the subsequent treatment.

For confirmed congenital gastrojejunal fistula, surgery is usually the standard and necessary treatment option. The patient reported in this article had postprandial abdominal pain for 6 months, but conservative treatment had no significant effect, indicating the need for surgery. The surgical principle involves identification and division of the abnormal fistulous tract followed by separate repair of the gastric and jejunal defects [11]. Conservative management is only applicable during the preoperative preparation phase to improve the patient’s nutritional status and general condition, thereby reducing surgical risks [12–14]. In rare circumstances where a congenital fistula remains asymptomatic and does not cause complications, careful observation with regular follow-up may be considered. Lifestyle modifications such as small frequent meals and avoiding the supine position after eating may help reduce reflux-related symptoms [15]. Nevertheless, most symptomatic patients require surgical correction. Surgical resection of the fistula with reconstruction of the gastrointestinal tract can effectively restore normal digestive physiology and prevent long-term complications such as ulcer perforation or dysplasia [16]. After multidisciplinary team discussion and thorough preoperative preparation, we decided to perform laparoscopic exploration in this case. Compared with conventional open surgery, laparoscopy offered advantages including less postoperative trauma, faster recovery, reduced intra-abdominal adhesions, etc. Intraoperatively, severe adhesions and significant anatomical variations were observed in the upper abdominal cavity, which initially precluded identification of the fistula. Therefore, intraoperative gastroscopy was employed to rapidly and accurately localize the fistula, which was subsequently resected. The patient recovered smoothly postoperatively, resumed a liquid diet on postoperative day 3, and was discharged on postoperative day 5. During the 6-month follow-up period, no specific discomfort or adverse events were reported.

In summary, this is the first report of case with congenital gastrofundic jejunal fistula in an adult, which is extremely rare and may remain undiagnosed for decades. Accurate diagnosis relies on comprehensive imaging and endoscopic evaluation. Although conservative management may be considered in exceptional asymptomatic cases, surgical intervention remains the preferred curative treatment for symptomatic patients. Individualized treatment decisions should be based on thorough clinical evaluation and risk assessment.

## Supplementary Material

carechecklist_rjag624
